# Brain regions associated with periodic leg movements during sleep in restless legs syndrome

**DOI:** 10.1038/s41598-020-58365-0

**Published:** 2020-01-31

**Authors:** Tae-Joon Kim, Kwang Su Cha, Sanghun Lee, Tae-Won Yang, Keun Tae Kim, Byeong-Su Park, Jin-Sun Jun, Jung-Ah Lim, Jung-Ick Byun, Jun-Sang Sunwoo, Jung-Won Shin, Kyung Hwan Kim, Sang Kun Lee, Ki-Young Jung

**Affiliations:** 1 Department of Neurology, Seoul National University Hospital, Seoul, Republic of Korea; 2 Department of Neurology, Ajou University School of Medicine, Suwon, Republic of Korea; 3 Department of Neurology, Gyeongsang National University Changwon Hospital, Gyeongsang National University School of Medicine, Changwon, Republic of Korea; 4 Department of Neurology, Keimyung University Dongsan Medical Center, Daegu, Republic of Korea; 5 Department of Neurology, Ulsan University Hospital, Ulsan, Republic of Korea; 6 Department of Neurology, Kangnam Sacred Heart Hospital, Hallym University College of Medicine, Seoul, Republic of Korea; 7 Department of Neurology, Kyung Hee University Hospital at Gangdong, Seoul, Republic of Korea; 8 Department of Neurosurgery, Seoul National University Hospital, Seoul, Republic of Korea; 9 Department of Neurology, CHA Bundang Medical Center, CHA University, Seongnam, Republic of Korea; 10 Department of Biomedical Engineering, College of Health Science, Yonsei University, Wonju, Republic of Korea; 11 Department of Neurology, Chamjoeun Hospital, Gwangju, Republic of Korea; 12 Department of Neurology, Seoul National University College of Medicine, Seoul, Republic of Korea

**Keywords:** Central pattern generators, Sleep disorders, Sleep disorders

## Abstract

The neural substrates related to periodic leg movements during sleep (PLMS) remain uncertain, and the specific brain regions involved in PLMS have not been evaluated. We investigated the brain regions associated with PLMS and their severity using the electroencephalographic (EEG) source localization method. Polysomnographic data, including electromyographic, electrocardiographic, and 19-channel EEG signals, of 15 patients with restless legs syndrome were analyzed. We first identified the source locations of delta-band (2–4 Hz) spectral power prior to the onset of PLMS using a standardized low-resolution brain electromagnetic tomography method. Next, correlation analysis was conducted between current densities and PLMS index. Delta power initially and most prominently increased before leg movement (LM) onset in the PLMS series. Sources of delta power at −4~−3 seconds were located in the right pericentral, bilateral dorsolateral prefrontal, and cingulate regions. PLMS index was correlated with current densities at the right inferior parietal, temporoparietal junction, and middle frontal regions. In conclusion, our results suggest that the brain regions activated before periodic LM onset or associated with their severity are the large-scale motor network and provide insight into the cortical contribution of PLMS pathomechanism.

## Introduction

Periodic leg movements during sleep (PLMS) are repetitive, involuntary, and jerky movements that typically involve dorsiflexion of the ankle, extension of great toe, and occasional flexion of the knee and hip^1^. The uniqueness of PLMS is that they occur periodically at intervals of approximately 20 to 40 seconds and usually in non-rapid eye movement (non-REM) sleep stages and are mostly presented in the legs. Previous studies investigating the PLMS pathophysiology revealed that changes in electroencephalography (EEG) accompanied by an increase in heart rate precede the occurrence of leg movements (LMs) during PLMS and that cortical and autonomic activations are regarded to lead these changes^2–8^, supporting the association between PLMS and cardiovascular risks^9^. However, which brain areas or neural substrates are involved in triggering PLMS have not been investigated.

Neural substrates involved in voluntary motor movements have been extensively studied using electrophysiological, neuroimaging, or hemodynamic studies^10–12^. Readiness potential, an initial slow potential shift in EEG, begins approximately two seconds before voluntary motor movement onset in the contralateral supplementary motor area. This feature also precedes the self-intention to move^12^. Areas associated with voluntary motor control include the dorsal premotor cortex and precuneus bilaterally and the left dorsolateral prefrontal, left inferior parietal, and right posterior cingulate regions^13,14^.

Because PLMS is an involuntary movement, we hypothesized that its brain areas involved would be similar to those of voluntary motor control except for the areas relevant to self-intention^15–17^. Based on the previous literature, neuronal pathways involved in PLMS may connect the self-originating region and supplementary motor area with the corticospinal tract^18^. The cerebral region associated with the first EEG changes observed prior to the start of PLMS may trigger PLMS. In addition, we hypothesized that there would be a region associated with severity of PLMS. The severity of PLMS can be investigated and represented by the association with parameters such as the PLMS index (PLMSI)^19,20^.

The aims of the present study were (1) to identify brain regions associated with intracerebral oscillatory activity preceding PLMS and (2) to find neural substrates related to the severity of PLMS at a sub-lobar level. To address these issues, the brain regions related to PLMS were identified by applying standardized low-resolution brain electromagnetic tomography (sLORETA)^21^, an EEG source localization method, from the initial EEG changes prior to PLMS onset. Brain regions specifically associated with PLMS were investigated by comparing the EEG sources of periodic LM (pLM) with those of isolated LM (iLM). The severity of PLMS was investigated by correlation with PLMSI.

## Methods

### Participants

We retrospectively reviewed and collected demographic information, sleep questionnaires, and polysomnographic (PSG) data from 19 drug-naïve RLS patients who participated in a previous study^22^. Participants were between the ages of 22 and 69 years old (median, 50 years old) and were recruited into the study between Mar 2012 and Feb 2013. RLS was diagnosed according to the diagnostic criteria proposed by the International RLS Study Group utilizing the validated Korean-language version of the Johns Hopkins Telephone Diagnostic Questionnaire^23,24^. The inclusion and exclusion criteria were described previously^22^. The questionnaires included the Epworth Sleepiness Scale, the Insomnia Severity Index, the Pittsburgh Sleep Quality Index, and the Korean version of Beck Depression Inventory II. The International RLS Study Group rating scale (IRLS) was applied to determine RLS severity. Two participants were excluded from the study because they had no PLMS. Another two participants were excluded from the subsequent data analysis due to poor quality of the PSG data. As a result, the final analysis included 15 subjects. This work was approved by the Institutional Review Board of Seoul National University Hospital (IRB no. 1705-118-855) and informed consent was obtained from all participants. The experimental protocol was carried out in accordance with the guidelines and regulations of the local ethics committee.

### Polysomnographic recordings

All participants underwent one night of PSG (Embla RemLogic; Embla Systems LLC, Broomfield, CO, USA) according to standard protocols as previously described^25^. Overnight PSG data were recorded using 19 EEG electrodes placed on the scalp in accordance with the international 10–20 system, two electrooculography channels, an electromyogram (EMG) of submental and anterior tibialis muscles, and an electrocardiogram (ECG) with surface electrodes. EEG signals were sampled at 200 Hz. Sleep stages were scored in 30-second epochs according to the standard criteria listed in the American Academy of Sleep Medicine sleep scoring manual^26^. We obtained total sleep time, sleep efficiency, sleep stages (including N1, N2, N3, and REM sleep), sleep latency, wake-up after sleep onset, apnea-hypopnea index, and arousal index via PSG. Each sleep stage was defined as a percentage of total sleep time.

### LM and PLMS measures

LM and PLMS were manually scored according to the World Association of Sleep Medicine/International RLS Study Group criteria^27^. LM was defined as any anterior tibialis EMG event with the criteria that the onset has EEG activity ≥8 μV in amplitude above resting baseline, the offset is under 2 µV, and the duration is ≤0.5 second. PLMS were defined as a series of four or more consecutive candidate LMs lasting 0.5–10 seconds and separated by intervals of 10–90 seconds during sleep.

PLMSI was defined as the average number of LMs included in PLMS per hour. Inter-movement interval (IMI) indicated the duration between the onset of one LM and the onset of the following LM^19^. The number of IMIs that were 10–90 seconds long and in sequences of ≥3 was divided by the total number of intervals to yield the periodicity index (PI); this index can vary between 0 (absence of periodicity, with none of the intervals between 10 and 90 seconds long) and 1 (complete periodicity, with all intervals between 10 and 90 seconds long).

In the present study, pLM indicated an LM that belonged to a PLMS series, and iLM indicated an LM that did not belong to a PLMS series.

### LM selection and EEG preprocessing

LMs only during non-REM sleep stages were included in the analysis. EEG data were processed using a band-pass filter (1–80 Hz) and a notch filter (60 Hz) to reduce background noise. EEG, EMG, and ECG data were segmented for −20~20 seconds around LM onset. LMs that had another LM in the preceding interval (−20~0 seconds) were rejected to exclude fluctuations in the baseline due to the preceding LM. LMs having signal fluctuations in the preceding period were also excluded by visual inspection. EEG segments exceeding an absolute amplitude of 150 μV were excluded from the analysis. EEG segments with any significant artifacts were also rejected by visual inspection. Independent component analysis was performed to remove EEG signal fluctuations, such as eye movement and skin potential, from the original data.

### Time-frequency analysis of EEG in relation to LM

To identify event-related changes in specific EEG spectral characteristics across LM conditions, the time-varying spectral power was estimated using short-time Fourier transform (STFT) with the following parameters: Hanning window and a 1-second time interval with a non-overlap. We did not overlap the time window for clarifying the instantaneous power changes. The magnitudes of the STFT coefficient were corrected with that of the baseline interval according to the following formula: Baseline-normalized power = (power − average power of baseline interval)/(standard deviation of power of baseline interval).

**Figure 1:**
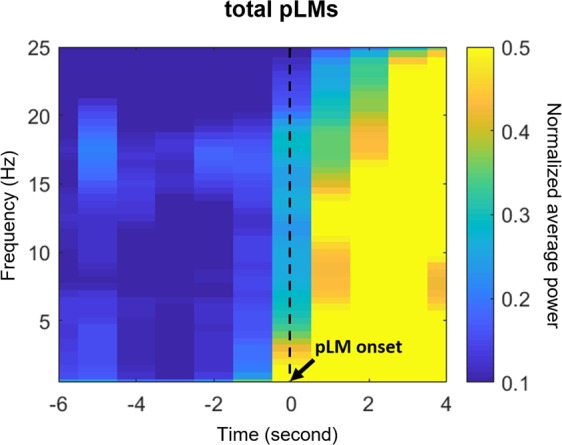
Time-frequency maps of power near the pLMs. Power was averaged over all electroencephalographic channels. Abbreviations: pLM, LM belonging to PLMS; LM, leg movement; PLMS, periodic leg movements during sleep.

The baseline interval was defined in this study as −18 to −12 seconds prior to the onset of LM. The baseline-normalized power was averaged across trials, that is, a total of pLMs or iLMs from all subjects. EEG spectra were divided into delta (2–4 Hz), theta (4–8 Hz), alpha (8–13 Hz), and beta (16–30 Hz) bands. Delta-band power preceded the other spectral powers and showed the most significant and remarkable transitions before PLMS (Fig. 1), in accordance with previous studies^28–30^. Therefore, we focused on delta bands in further analyses. Topographic maps are presented for each time point according to each frequency band to depict the overall temporal and spatial power distributions. The average values of spectral power across all EEG channels were used to compare signals obtained from different kinds of LM.

To differentiate EEG changes associated with PLMS from those associated with spontaneous EEG arousal not accompanied by any other event, spectral analysis of spontaneous EEG arousal was performed using the same method used for LMs.

### Heart rate changes

The time interval between two successive enhanced R-peaks was used to calculate heart rate. To obtain more highlighted R-peaks, band-pass filtering was conducted. The signal was filtered by fourth-order Butterworth filters with cut-off frequencies between 15 and 20 Hz. The heart rate was computed from the R-R interval, and the baseline-normalized heart rate was obtained using the same formula as that used for baseline-normalized power.

### EEG source localization

From the scalp-recorded EEG potentials, a three-dimensional reconstructed source distribution for the delta-band activity beginning prior to PLMS onset was obtained using the sLORETA method implemented in Brainstorm version 3.2, an open source toolbox^31^. The solution space was restricted to the cortical gray matter in 15,002 voxels with a spatial resolution of 1 mm in the Talairach coordinates^32^. To group the voxels into functionally similar cortical regions, we employed a total of 148 regions of interest (ROIs) adopted from a standard magnetic resonance imaging (MRI) template obtained from Destrieux *et al*. This ROI method was implemented for comparing normalized power between pLM and iLM or for correlating current densities with PLMSI. The principal component analysis was performed to determine the dominant activity among all voxels at each ROI. At each ROI, current density was standardized compared to that in the baseline to test for statistical significance.

### Statistical analysis

Clinical data are presented as the medians and 25th–75th interquartile ranges. EEG spectral power or heart rate data at every time point near LM onset were compared with the average of those obtained during the baseline interval using paired samples t-tests. An uncorrected p value of less than 0.01 was considered statistically significant to study data from scalp-recorded EEG channels or ECG heart rate. For source localization, a t-distribution was adopted to show brain regions that were significantly activated compared with the activity observed at baseline. To compensate for multiple comparisons when investigating the EEG sources, the false discovery rate (FDR) was applied to the statistically significant regions of t-distribution versus baseline (FDR-adjusted p value < 0.005)^33^. A correlation analysis between clinical scores, including IRLS, ferritin levels, or PLMSI, and current density for EEG delta power, was performed using Pearson’s correlation, and p values of less than 0.05 were considered statistically significant. The data analyses and statistical analyses were performed using MATLAB software version R2014b (Mathworks, Inc., Natick, MA, USA).

## Results

### Clinical characteristics of RLS patients

The median age of the 15 subjects was 52 [25–75% interquartile range; 40–58] years old, and 13 of them were female. Their epidemiological characteristics, sleep questionnaire scores, and PSG parameters are summarized in Table 1. The median PLMSI was 10.5 [6.5–35.1], and the median value of the mean IMI in PLMS was 36.7 [32.9–39.9]. The total number of pLMs identified in the PSG of 14 subjects was 1,871. Of these, 274 pLMs (14.6%) were excluded according to the condition having preceding another pLM within 20 seconds, and 47 ones were excluded due to artifacts. Therefore, a total of 1,550 pLMs obtained from 14 subjects and 251 iLMs obtained from another 14 subjects were subjected to subsequent analyses.

**Table 1:** Clinical information and polysomnography data of all 15 subjects included in the analysis.

Item	Median [25–75% interquartile range] or percentage
Age (year)	52 [40–58]
Sex (female %)	86.7%
BMI (kg/m^2^)	22.6 [21.1–23.3]
RLS features	
Symptom duration (years)	20 [10–25]
Symptom frequency (/week)	6.5 [4.5–6.5]
IRLS score	32 [27–34]
Ferritin (ng/mL)	128.8 [23.8–172.6]
Questionnaires	
ESS	6 [4–9]
ISI	20 [15–24]
PSQI	15 [9–17]
BDI-II	16 [9–27]
PSG parameters	
TIB (min)	432 [404–447]
TST (min)	352 [311–386]
SE (%)	83.3 [70.3–91.3]
SL (min)	9.0 [2.8–26.5]
WASO (min)	56.3 [32.5–86.9]
N1 (%)	17.1 [13.9–25.2]
N2 (%)	38.7 [33.4–49.0]
N3 (%)	16.9 [10.4–25.4]
R (%)	19.2 [16.6–22.0]
AHI (/hr)	1.5 [0.4–6.0]
Arousal index (/hr)	21.3 [17.4–25.9]
PLMSI (/hr)	10.5 [6.5–35.1]
PI	0.58 [0.40–0.72]
Mean IMI (s) in PLMS	36.7 [32.9–39.9]

- Abbreviations: BMI, body mass index; RLS, restless legs syndrome; IRLS, International RLS Study Group rating scale; ESS, Epworth Sleepiness Scale; ISI, Insomnia Severity Index; PSQI, Pittsburgh Sleep Quality Index; BDI-II, Beck Depression Inventory II; PSG, polysomnography; TIB, time in bed; TST, total sleep time; SE, sleep efficiency; SL, sleep latency; WASO, wake-up after sleep onset; AHI, apnea-hypopnea index; PLMSI, periodic leg movement during sleep index; PI, periodicity index; IMI, inter-movement interval.

### Changes in heart rate and EEG spectral power preceding LM onset

**Figure 2:**
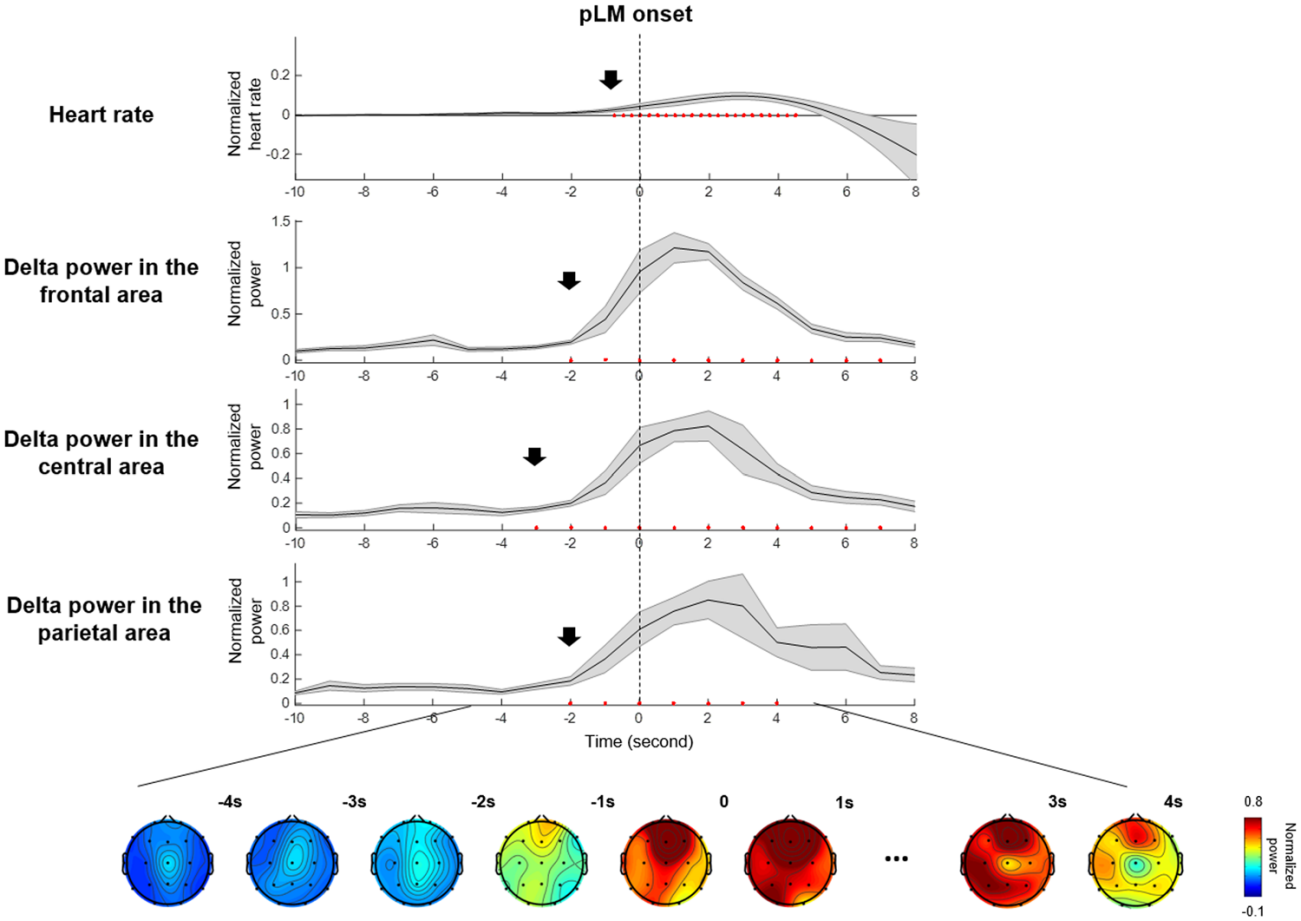
Delta-band (2–4 Hz) power at three areas, topography, and heart rate changes near pLM onset. Delta-band power in the central area increased significantly at −3~−2 seconds before pLM onset. Heart rate increased at −1 second, and delta power in the frontal and parietal areas increased at −2~−1 seconds before pLM onset. Red dots indicate significant changes compared to the value of baseline interval using paired t-test (uncorrected p < 0.01). An EEG power value at −10 seconds indicates normalized power at −10~−9 seconds, and so on. Abbreviations: pLM, LM belonging to PLMS; LM, leg movement; PLMS, periodic leg movements during sleep.

EEG and ECG signals changed before pLM onset, in line with previous studies. The heart rate began to increase significantly at −1 second before pLM onset. Averaged spectral power over 19 EEG channels increased significantly at around −2 seconds before pLM onset (p < 0.01 compared with the baseline interval) in all frequency bands, especially beta and delta bands (Fig. 1). In particular, the delta-band power was the most initial and prominent signal to increase (Fig. 2). The delta-band power in the central area was the first signal to increase at −3~−2 seconds prior to pLM in PLMS and was followed by signals in the frontal and parietal areas. Beta-band power was the second prominent signal following delta band and began to increase in the frontal area at −2~−1 seconds (Fig. S1). iLM showed slightly different patterns of changes in EEG spectral power from those of the pLM. Delta power prior to iLM onset increased in the frontal area at −3~−2 seconds followed by in the central and parietal areas (Fig. S2).

### EEG sources associated with PLMS

Because the delta band was the most initial and prominent signal before pLM onset at approximately −3 seconds, further analysis employed the delta band to assess the brain regions associated with PLMS. We identified the EEG current sources for the delta bands preceding PLMS using the sLORETA method.

**Figure 3:**
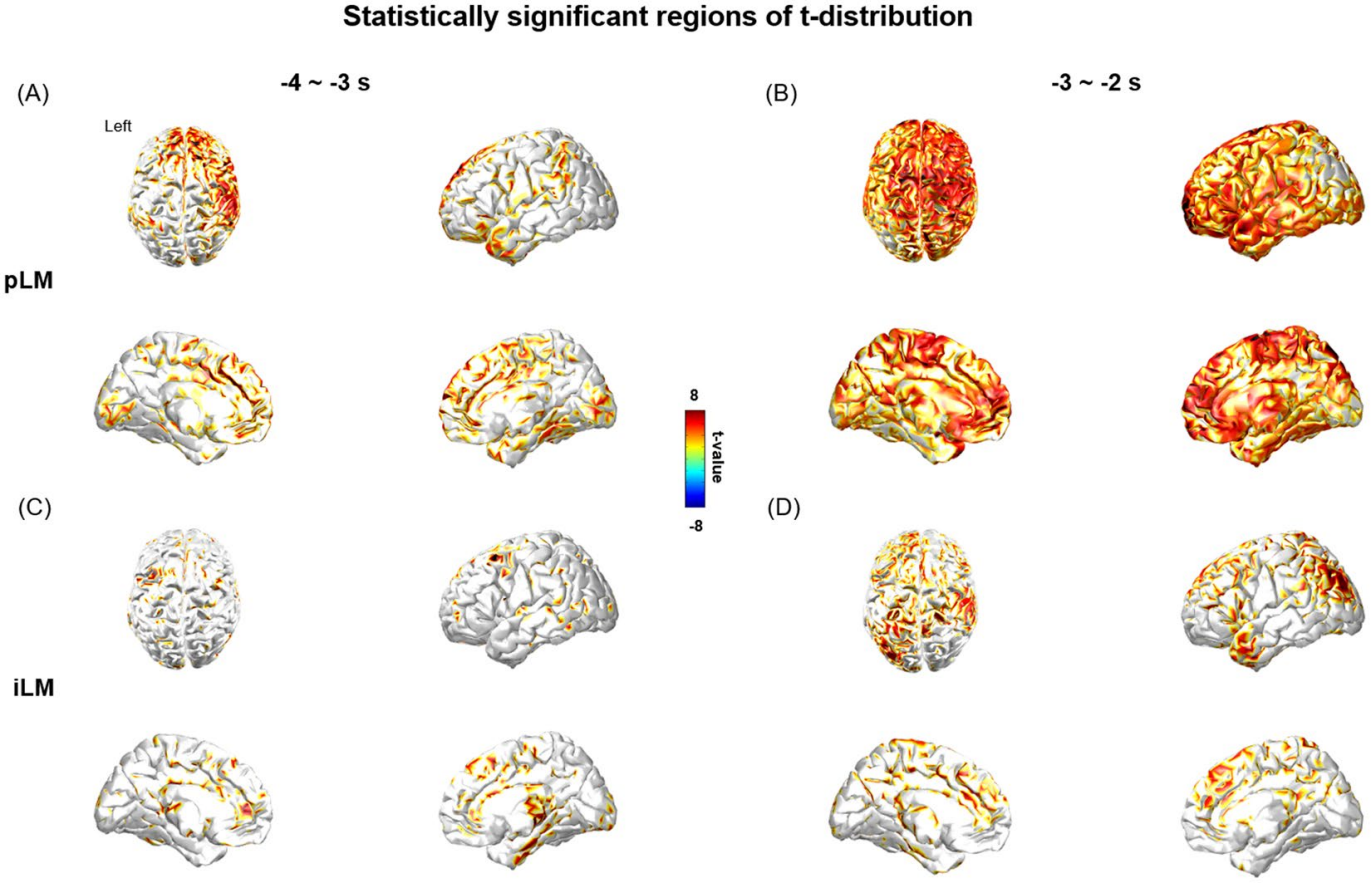
Statistically significant regions of t-distribution of delta-band current sources before pLM or iLM onset versus baseline (FDR correction, FDR-adjusted p value < 0.005) were demonstrated. (**A**) The significant regions at −4~−3 seconds before pLM onset were right pericentral, bilateral dorsolateral, and cingulate regions compared to the baseline interval, and (**B**) spreads diffusely over fronto-temporal lobes at −3~−2 seconds. (**C**) In contrast, the significant regions were sparsely observed at −4~−3 seconds before iLM onset. (**D**) At −3~−2 seconds before iLM onset, the significant regions were mainly in the right pericentral and left parieto-occipital areas. Abbreviations: pLM, LM belonging to PLMS; iLM, LM not belonging to PLMS; LM, leg movement; PLMS, periodic leg movements during sleep; FDR, false discovery rate.

Several brain regions, including the bilateral pericentral, dorsomedial prefrontal, and posterior cingulate regions, had higher EEG current densities before pLM onset (Fig. S3A,B). In contrast, cortical activity before iLM onset began to activate in the bilateral dorsal, ventrolateral, medial frontal, and anterior cingulate regions (Fig. S3C,D). Statistically significant current sources of delta power emerged first at -4 ~ -3 seconds before pLM onset and were mainly located in the right pericentral, bilateral dorsolateral prefrontal, and cingulate regions (Fig. 3A). The current sources then spread out into the bilateral frontal and temporal areas with time (Fig. 3B). Current sources of delta power before iLM onset were sparsely detected until −4~−3 seconds (Fig. 3C) and after −3~−2 seconds appeared at the areas encompassing the right pericentral and left parieto-occipital regions (Fig. 3D).

### Correlation between PLMSI and cortical activity

**Figure 4:**
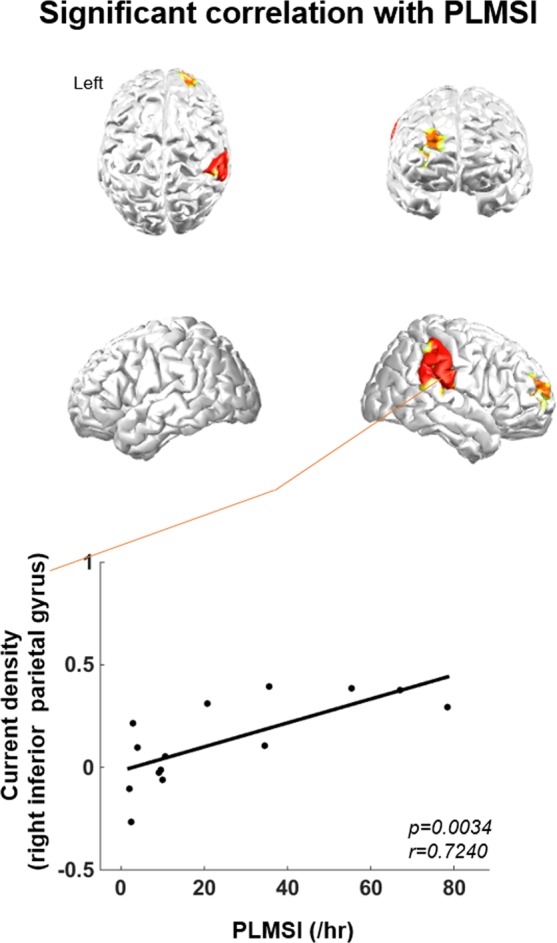
Correlation between PLMSI and cortical activity. At the right inferior parietal, right middle frontal region, and superior temporal sulci, delta current densities between −3~−2 seconds before pLM versus baseline interval had significant positive correlation with PLMSI. Abbreviations: pLM, LM belonging to PLMS; LM, leg movement; PLMS, periodic leg movements during sleep; PLMSI, PLMS index.

Considering that the significant EEG sources before pLM emerged initially at between −4~−3 or −3~−2 seconds, the cortical activity represented by the current density of delta bands during these time intervals was correlated with PLMSI. Brain regions that had significant correlations with PLMSI were the right inferior parietal, right middle frontal region, and superior temporal sulci at −3~−2 seconds (Fig. 4). No brain regions were correlated with IRLS or ferritin levels.

## Discussion

The results of our study reveal that the brain regions associated with PLMS were the large-scale motor network, including pericentral, dorsolateral prefrontal, and cingulate regions, at −4~−3 seconds before each repetitive LM. We also showed that part of default mode network and motor control area, comprising the right inferior parietal, temporoparietal junction, and middle frontal regions, had significant correlations with PLMSI. These results provide new insights into the neural substrates in relation to PLMS in RLS.

We first confirmed that EEG activity preceded LM by a few seconds, in line with the literature. This finding was observed in wide spectral bands from the delta to the beta band, but the delta band was the initial signal that showed the most pronounced increase. Delta activity has been known to be the main spectrum preceding motor movement and is understood to be the signal most related to motor initiation and preparation^28–30^. For these reasons, we analyzed the delta bands for EEG source localization in the present study.

EEG topographic data of the delta band showed that the pericentral area was the first to change before pLM onset and that the frontal and parietal areas followed afterwards. In the next step, the EEG current source of the delta band was determined using the sLORETA method, which identified the specific brain regions that were activated before PLMS. As a result, we confirmed that the neural substrates preceding pLM were localized at the pericentral, dorsolateral prefrontal, and cingulate regions. The dorsolateral prefrontal cortex has various functions, including motor control^13^. The posterior cingulate cortex, as a central node in the default mode network, plays a role in executive motor control in connection with the frontoparietal control network^34^. A positron emission tomography study demonstrated that the bilateral pericentral and right posterior cingulate regions corresponded to the sites activated during motor imagery in locomotor-related tasks^13^. Increasing evidence in neuroimaging and neuroanatomical studies supports the idea that some subset of anterior cingulate motor area neurons are involved in motor control preparation and execution^35–37^. The white matter volume of the anterior cingulate cortex is smaller in patients with RLS^38–40^. A functional MRI (fMRI) study revealed that the left primary motor and somatosensory cortices with ventral anterior cingulum were activated during periodic leg movements in RLS patients^41^. Therefore, the activated brain regions associated with PLMS in RLS patients constitute the large-scale motor network, including the sensorimotor cortex with motor control regions, and mostly in agreement with the areas associated with voluntary movement.

It is important to consider the possibility that brain regions associated with PLMS are specific to PLMS or represent only an EEG arousal response. LMs in PLMS are sometimes accompanied by arousal, and in some previous reports in the literature, PLMS has been considered a kind of arousal response^2,8,42^. In this regard, it is necessary to confirm that the EEG source of this study is not simply caused by arousal; therefore, we evaluated spectral power associated with spontaneous arousal during sleep. The delta power associated with spontaneous arousal, which was not associated with any other events, was initially observed at the medial frontal area at −2 ~ 0 seconds and spanned over the left temporal area at 0~2 seconds (Fig. S4). The topographic data obtained for PLMS were initially different but overlapped with the sites activated during spontaneous arousal: the spectral power of the delta band in the central area initially increased at −3 ~ -2 seconds before PLMS, and subsequently, the medial frontal area was activated at −2 ~ -1 seconds (Fig. 2). This medial frontal activation in PLMS may indicate the late arousal response after motor network activation. Hence, the sources of PLMS found in this study were more associated with motor initiation rather than a related arousal response in agreement with studies by Ferri *et al*.^43^.

Neural substrates related to the severity of PLMS are proposed in the present study including inferior parietal, temporoparietal junction, and middle frontal regions. For example, subjects with more delta activity in the right inferior parietal region tended to have higher PLMSI (Fig. 4). Thus, these cortical regions are likely to be the neural substrates underlying the severity of PLMS. Increasing evidence in neuroimaging and neuroanatomical studies supports the idea that inferior parietal lobule as the default mode network is involved in motor control preparation and execution^13,44,45^. Interestingly, aforementioned fMRI study found that multiple areas, including the right inferior parietal lobule, were activated in periodic leg movements during wakefulness in RLS patients^41^. The results of the present study suggest that the default mode network with prefrontal motor control pathway may play a role in PLMS. In addition, the regions related to the severity were mainly observed in the right hemisphere, possibly due to hemispheric functional lateralization. Previous studies have shown that the right hemisphere has a specific role in motor control and that there is a right hemisphere preference for the control of slow repetitive movement^46,47^. From our results, it can be suggested that the severity in PLMS might be more associated with the right hemisphere.

Structural MRI data in RLS patients need to be reassessed because cortical structural changes have been mostly investigated in RLS patients. Previous studies using diffusion images or voxel-based morphometry in RLS patients in part support our results of brain regions associated with PLMS. Diffusion tensor imaging of RLS patients revealed that reduced fractional anisotropy was observed in the subcortical areas close to the motor and the somatosensory cortices as well as parts of the limbic system^40^. Connor et at. found decreases in white matter volume in RLS patients in the corpus callosum, anterior cingulum, and precentral gyrus using postmortem imaging analysis^39^. In another study with 28 RLS patients, the same group of researchers found decreases of cortical thickness in bilateral postcentral gyrus and corpus callosum posterior midbody indicating somatosensory pathway^48^. Interestingly, contrary to previous studies, recent multimodal MRI study by Stefani *et al*. with 87 RLS patients identified increases of gray matter volume of primary sensory cortex bilaterally, the left premotor cortex, and the right parietal inferior lobe which would fit to the concept of adaptive cortical plasticity as a result of increased neuronal activation during PLMS^49^. Further studies are needed to investigate the implication and causal relationship of a cortical role in PLMS with RLS.

Our results have clinical implications in terms of therapeutic targets for neuromodulatory treatment. Various neuromodulatory therapies, including transcranial direct current stimulation and repetitive transcranial magnetic stimulation, have been applied in patients with RLS^50–53^. One of the most important points to be considered is the therapeutic target to which stimulation should be given. Most studies have given stimulation on the primary motor cortex or supplementary motor area. EEG sources found in the present study may provide insight for new therapeutic targets for neuromodulation in the treatment of RLS with PLMS.

The present study needs to be interpreted in light of several limitations, as follows. First, the number of RLS patients analyzed in this study was small because PSG equipped with 19-channel EEG cannot be routinely performed. Further research with a larger number of patients using extended EEG channels is needed to support our results and the precise role and association of cortical contribution of PLMS. Second, the EEG source localization was relatively sparse because of the limitations inherent to our study methods. The number of EEG electrodes was not high (19 channels), and the standard electrode location coordinates and standard MRI template were applied without using individual data. Third, the role of subcortical regions during PLMS could not be evaluated in the present study, which was based on the electrophysiological analysis of cortical signals. Stereo-EEG studies, if researchers have opportunities such as presurgical evaluations using intracranial electrodes in patients with epilepsy, would therefore be helpful. Fourth, in the present study, PLMS was evaluated only in RLS patients, and various conditions associated with PLMS may have different characteristics^54^. Thus, whether the brain regions associated with PLMS in the present study can be generalized to other conditions showing PLMS remains unclear. EEG sources in PLMS need to be validated in other disorders^18^. Finally, pLMs with short intervals of less than 20 seconds were excluded from the analysis due to baseline interval setting, which may have affected the overall results.

Our study suggests that the large-scale motor network may be involved in initiating PLMS in RLS. These factors contribute to our understanding of the pathophysiology of PLMS. Further validation with other neuroimaging investigations is warranted to delineate this association better and to increase our understanding of the pathogenesis of PLMS.

## Supplementary information

Supplementary Figures.

## Data Availability

Polysomnographic signals and preprocessed data analyzed during the current study are not publicly available due to compliance to privacy. Summary statistics are available from the corresponding author on reasonable request.
