# Supplementary material for: Brain regions associated with periodic leg movements during sleep in restless legs syndrome
Source: Sci Rep. 2020 Jan 31;10:1615. doi: 10.1038/s41598-020-58365-0 (PMC6994717; doi:10.1038/s41598-020-58365-0)
Supplement: Supplementary file 1 — Supplementary Figures. [file 41598_2020_58365_MOESM1_ESM.docx]

**Supplementary Information**

**Brain regions associated with periodic leg movements during sleep in restless legs syndrome**

Tae-Joon Kim^1,2,*^, Kwang Su Cha^1,*^, Sanghun Lee^1^, Tae-Won Yang^3^, Keun Tae Kim^4^, Byeong-Su Park^5^, Jin-Sun Jun^6^, Jung-Ah Lim^1^, Jung-Ick Byun^7^, Jun-Sang Sunwoo^8^, Jung-Won Shin^9^, Kyung Hwan Kim^10^, Sang Kun Lee^1¶^, Ki-Young Jung^1¶^

^1^Department of Neurology, Seoul National University Hospital, Seoul, Republic of Korea

^2^Department of Neurology, Ajou University School of Medicine, Suwon, Republic of Korea

^3^Department of Neurology, Gyeongsang National University Changwon Hospital, Gyeongsang National University School of Medicine, Changwon, Republic of Korea

^4^Department of Neurology, Keimyung University Dongsan Medical Center, Daegu, Republic of Korea

^5^Department of Neurology, Ulsan University Hospital, Ulsan, Republic of Korea

^6^Department of Neurology, Kangnam Sacred Heart Hospital, Hallym University College of Medicine, Seoul, Republic of Korea

^7^Department of Neurology, Kyung Hee University Hospital at Gangdong, Seoul, Republic of Korea

^8^Department of Neurosurgery, Seoul National University Hospital, Seoul, Republic of Korea

^9^Department of Neurology, CHA Bundang Medical Center, CHA University, Seongnam, Republic of Korea

^10^Department of Biomedical Engineering, College of Health Science, Yonsei University, Wonju, Republic of Korea

^*^Co-first authors; these two authors contributed equally to the manuscript.

^¶^Co-corresponding authors; these two authors contributed equally to the manuscript.

**Supplementary Figure 1**


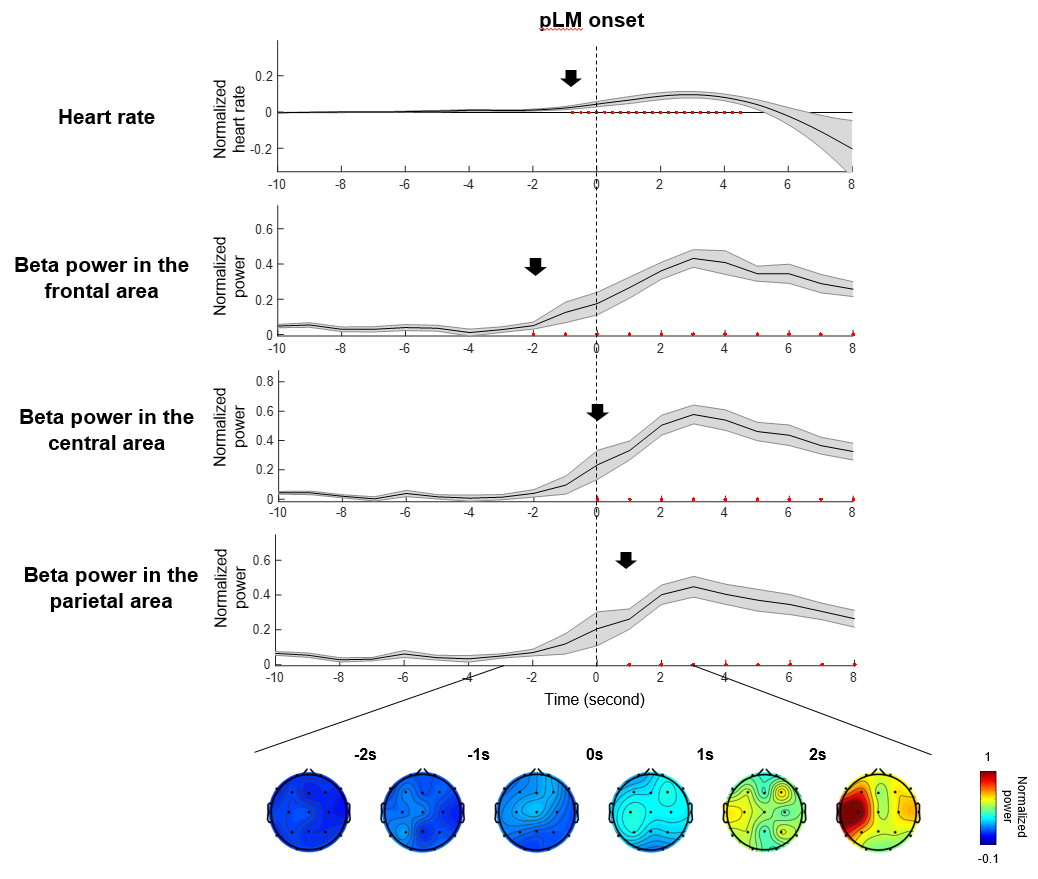


**Supplementary Figure 1.** Beta-band (16-30 Hz) power at three areas, topography and heart rate changes near pLM onset. Beta power in the frontal area increased significantly at -2 ~ -1 seconds before pLM onset. Red dots indicate significant changes compared to the value of baseline interval using paired t-test (uncorrected p < 0.01). An EEG power value at -10 second indicates normalized power at -10 ~ -9 seconds, and so on.

Abbreviations: pLM, LM belonging to PLMS; LM, leg movement; PLMS, periodic leg movements during sleep.

**Supplementary Figure 2**


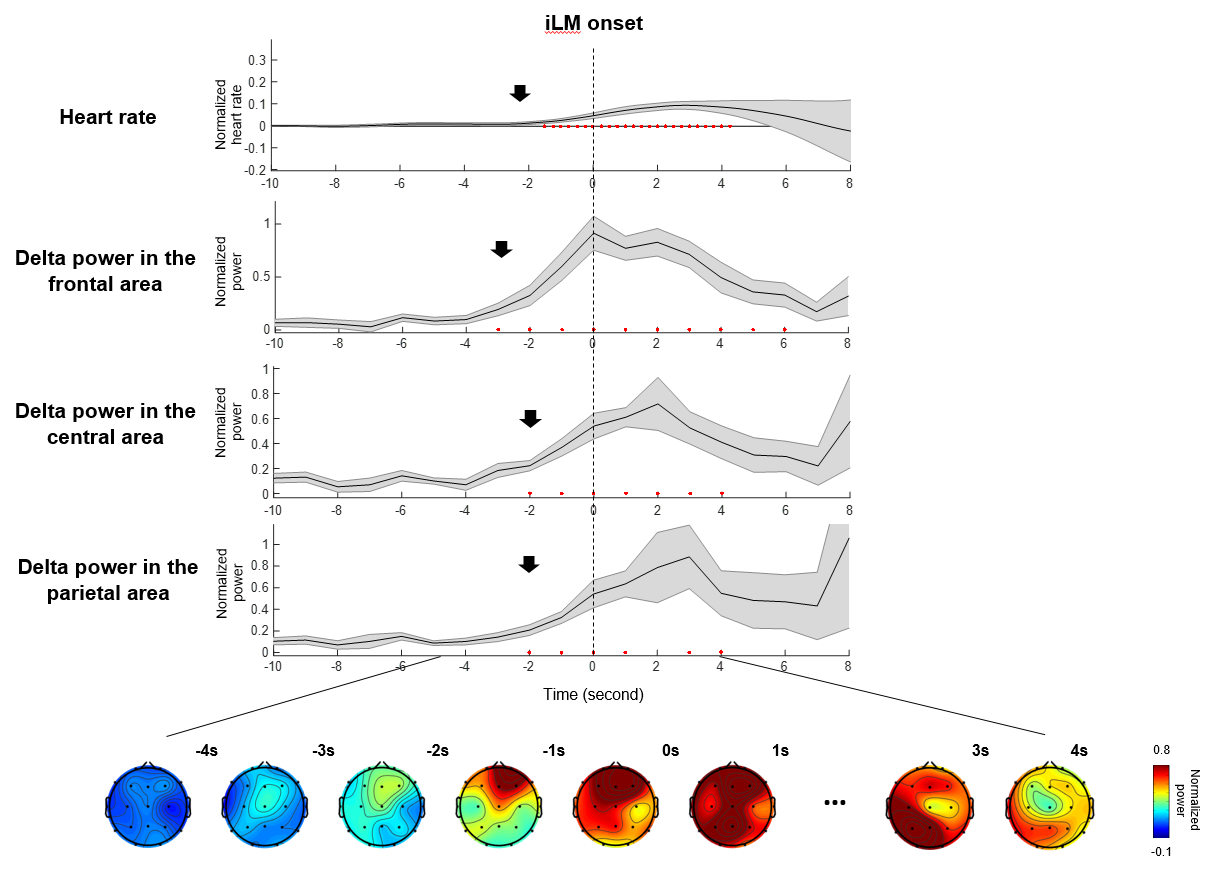


**Supplementary Figure 2.** Delta-band (2-4 Hz) power at three areas, topography, and heart rate changes near iLM onset. Delta power in the frontal area increased significantly at -3 ~ -2 seconds before iLM onset. Heart rate increased at -2 second, and delta power in the central and parietal areas increased at -2 ~ -1 seconds before iLM onset. Red dots indicate significant changes compared to the value of baseline interval using paired t-test (uncorrected p < 0.01). An EEG power value at -10 second indicates normalized power at -10 ~ -9 seconds, and so on.

Abbreviations: iLM, LM not belonging to PLMS; LM, leg movement; PLMS, periodic leg movements during sleep.

**Supplementary Figure 3**


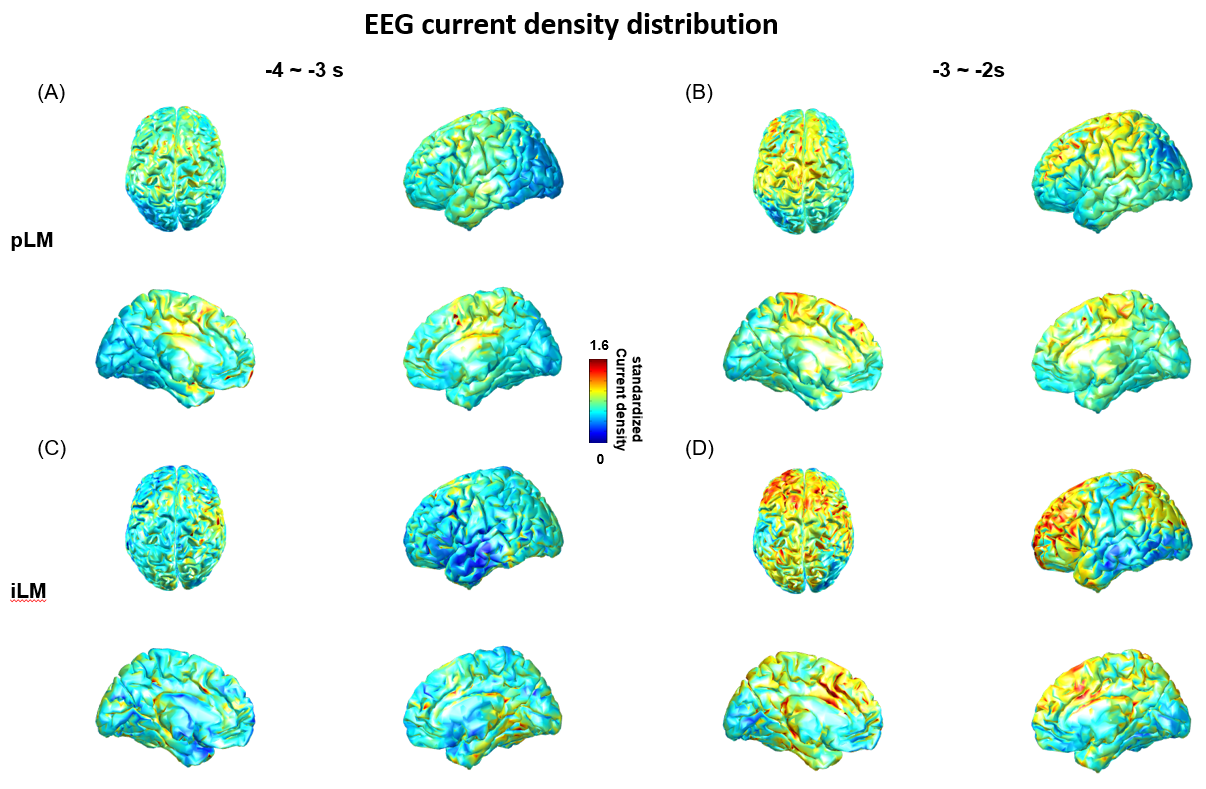


**Supplementary Figure 3.** EEG current sources of delta-band power at -4 ~ -3 and -3 ~ -2 seconds before pLM or iLM onset. The current sources before pLM were located mainly at the bilateral pericentral, dorsomedial prefrontal, and posterior cingulate cortices. By contrast, the current sources before iLM were located mainly at the bilateral dorsal, ventrolateral., and medial frontal areas and anterior cingulate cortices at -3 ~ -2.

Abbreviations: pLM, LM belonging to PLMS; iLM, LM not belonging to PLMS; LM, leg movement; PLMS, periodic leg movements during sleep.

**Supplementary Figure 4**


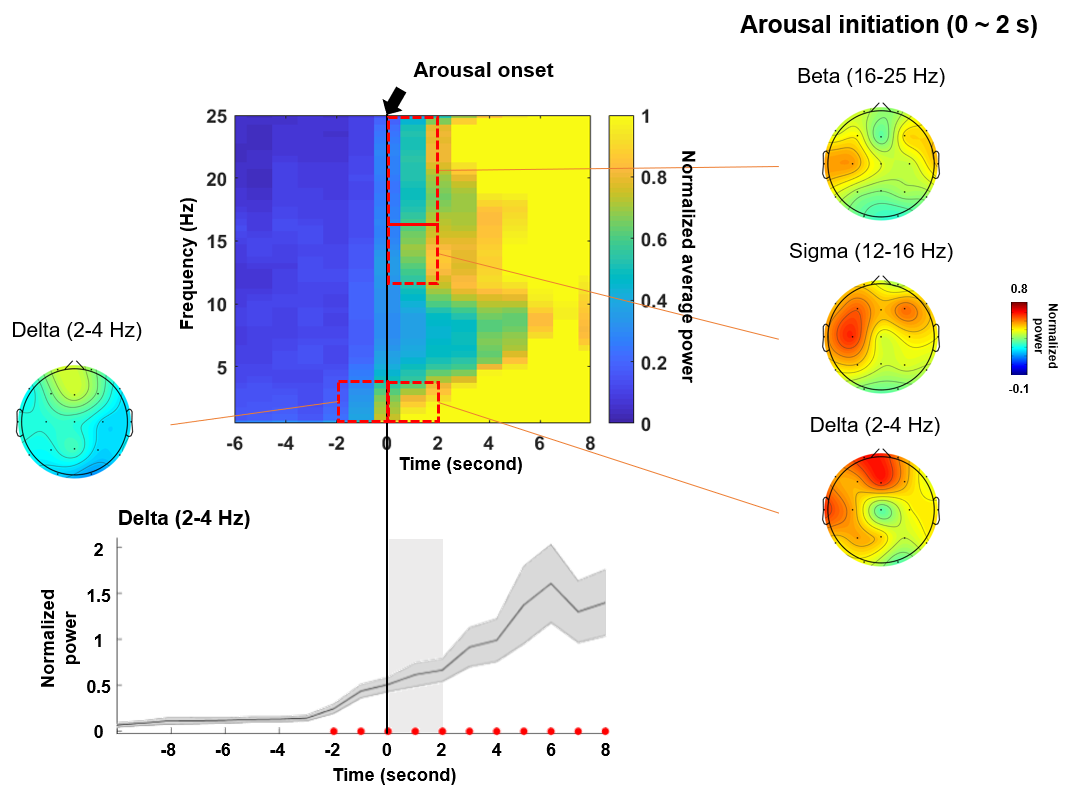


**Supplementary Figure 4.** Time-frequency map and delta power during spontaneous arousal. Delta power started to change mainly in the midfrontal area from -2 seconds before arousal onset. After arousal onset, midfrontal and left temporal delta power increased along with sigma and beta powers in the left temporal and bifrontal areas.
